# Clinical Cancer Genetics in a Lower-Middle Income Country: Considerations for Policymaking

**DOI:** 10.1200/JGO.18.00081

**Published:** 2018-07-06

**Authors:** Ophira Ginsburg, Steven A. Narod

**Affiliations:** **Ophira Ginsburg**, New York University Langone Health, New York, NY; and **Steven A. Narod**, Women’s College Research Institute, Toronto, Ontario, Canada.

The field of cancer genetics is entering the mainstream of clinical oncology. At least that is the case for many high-income—and some upper-middle income—countries. We hope that these developments will lessen the burden of cancer in countries with limited resources and will not divert funds that could be better invested elsewhere. In the article that accompanies this editorial, Adejumo et al^1^ present the results of a pilot study from Ibidan, Nigeria, that explores knowledge among patients with breast cancer and their close relatives of the role of heredity in breast cancer and of genetic counseling for breast cancer. Ninety percent of patients had heard of breast cancer, but few could describe the causes. Almost one fifth of patients believed that breast cancer was a kind of spiritual attack. Clinical genetics services, by and large, are unavailable in Nigeria, and it is not surprising that 91% of patients did not know anything about cancer genetics or genetic testing.

A comprehensive state-of-the-art program in clinical cancer genetics includes cancer risk assessment and genetic counseling and testing.^2^ The genetic counselor provides a detailed assessment of the patient’s family history of cancer and provides tailored, guidelines-based advice on cancer risk reduction for unaffected women with positive genetic test results. This information can also inform risk reduction for future cancers as well as precision treatment for patients with a current cancer diagnosis. Genetic testing has become increasingly complex with the adoption of multigene panels and the attendant flurry of variants of uncertain significance, often in genes of questionable importance. One wonders how many patients will benefit from these technical advances, whether in North America or Nigeria. We applaud efforts to modernize cancer prevention and treatment by increasing genetic knowledge around the world, but we ask the authors if it is wise to pursue such a study in any country in which the health system is fragile and fragmented. Nigeria, among the largest economies in sub-Saharan Africa, is classified as a lower-middle income country.^3^ Nigeria’s maternal mortality ratio is ranked as the fourth highest globally.^4,5^ According to the World Bank, in 2014 the country ranked among the lowest in terms of health expenditures in the public sector at 0.9% of gross domestic product.^6^ Out of pocket expenditures are estimated at 49.5%.

Editorialists should not make naïve assumptions about health financing priorities in any country, nor should we dictate how policymakers use their resources. We acknowledge that cancer genetics research has the potential to advance our understanding of genetics and may reduce global disparities in cancer incidence and mortality. In Nigeria, the pioneering work of Olopade et al^7^ has led to several important discoveries that are not only relevant for women in Nigeria but also for women of West African ancestry. A study of Nigerian women with breast cancer unselected for age or family history demonstrated that 7.1% tested positive for a pathogenic mutation in *BRCA1*, and 3.9% had *BRCA2* mutation; the 11% total for unselected women is among the highest rates of any population studied.^7^ To translate this knowledge into a reduced cancer burden will require systematic testing and appropriate follow-up of carriers and their at-risk relatives for clinical interventions, including breast screening and risk-reduction surgery (bilateral mastectomies and salpingo-oophorectomies). We should not deny the potential impact of such research when counseling and testing an individual and family can lead to life-saving cancer prevention and risk-reduction opportunities, and we should strive to make these options acceptable, available, and affordable. Of interest, the traditional paradigm of nondirective counseling may run into problems when there is little background knowledge of genes and disease and superstition persists. Adejumo and colleagues note these challenges to the traditional—Western—genetic counseling model. That study participants had limited knowledge of their family histories challenges the paradigm for how we identify individuals who may harbor a germline mutation in a high-risk cancer susceptibility gene. This issue, along with other social and cultural factors that make case ascertainment and cancer genetic counseling challenging, have been described in other populations, notably in Asia^8^ and the Carribbean.^9^

Factors that influences the acceptability, availability, and uptake of clinical cancer genetics services can be considered to fall under a few broad categories: knowledge of hereditary cancer among providers and the public; sociocultural factors; infrastructure; and ethical, financial, legal, and regulatory considerations.^10^ A recent study from Brazil, published in *Journal of Global Oncology*,^11^ explores these factors and provides recommendations for priorities in planning for comprehensive cancer genetics services. In 2015, ASCO highlighted the critical issues of quality assurance, informed consent, and patient privacy and rights, including protection from genetic discrimination, cancer genetics education among cancer care providers, and disparities in access to clinical genetics services.^12^ Adejumo and colleagues make a reasonable case for investing in clinical cancer genetics services in Nigeria. They call for pretest counseling with informed consent, post-test counseling with disclosure of high-quality results, and the promotion of evidence-based interventions, ideally within the public health system or at reasonable cost in the private sector.

## References

[1] Adejumo P Aniagwu T Oluwatosin A et al Knowledge of genetic counseling among patients with breast cancer and their relatives at a Nigerian teaching hospital J Glob Oncoldoi:10.1200/JGO.17.00158 10.1200/JGO.17.00158 PMC6223535 30084716

[2] National Cancer Institute Cancer genetics risk assessment and counseling (PDQ): Health professional version.

[3] DataBank Health Nutrition and Population Statistics, World Bank. http://datatopics.worldbank.org/health/available-indicators

[4] Central Intelligence Agency The World Fact Book: Maternal mortality rate.

[5] World Health Organization Maternal mortality in 1990-2015.

[6] United Nations Population FundHuman Development Report. http://hdr.undp.org/en/indicators/53906#

[7] Fackenthal JD, Zhang J, Zhang B (2012). High prevalence of BRCA1 and BRCA2 mutations in unselected Nigerian breast cancer patients. Int J Cancer.

[8] Nakmura S, Kwong A, Kim SW (2016). Current status of the management of hereditary breast and ovarian cancer in Asia: First report by the Asian BRCA Consortium. Public Health Genomics.

[9] Narod SA, Butler R, Bobrowski D (2018). Short report: Follow‐up of Bahamian women with a BRCA1 or BRCA2 mutation. Mol Genet Genomic Med.

[10] Fashoyin-Aje L, Sanghavi K, Bjornard K (2013). Integrating genetic and genomic information into effective cancer care in diverse populations. Ann Oncol.

[11] Folgueira MAAK, Maistro S, Teixeira N How should genetic counseling for ovarian cancer be implemented in a middle-income country? An insight based on the Brazilian scenario. J Global Oncol.

[12] Robson ME, Bradbury AR, Arun B (2015). American Society of Clinical Oncology policy statement update: Genetic and genomic testing for cancer susceptibility. J Clin Oncol.

